# *Histoplasma capsulatum*: An Unusual Case of Pericardial Effusion and Coarctation of the Aorta

**DOI:** 10.14740/jocmr2455w

**Published:** 2016-01-26

**Authors:** Sarah Sansom, Aditya Shah, Shoeb Hussain, Jami Walloch, Sampath Kumar

**Affiliations:** a Department of Internal Medicine, University of Illinois at Chicago/Advocate Christ Medical Center, Oak Lawn, IL, USA; b Department of Pathology, University of Illinois at Chicago/Advocate Christ Medical Center, Oak Lawn, IL, USA

**Keywords:** Histoplasma, Histoplasmosis, Adult congenital heart disease, Pericarditis, Aortic coarctation, Pericardial effusion

## Abstract

*Histoplasma capsulatum* is a fungus that is endemic in many parts of the world and can present with a wide variety of symptoms. Here we present a case of a previously healthy 19-year-old female who presented with shortness of breath. She was found to have a right lung mass and coarctation of the aorta on computed tomography imaging. Pathology revealed granuloma caused by *Histoplasma capsulatum*. She later developed massive pericardial effusion, requiring emergent pericardiocentesis. She was treated with anti-fungal therapy and recovered well. This case illustrates an unusual presentation of newly diagnosed coarctation of the aorta complicated by Histoplasma pericardial effusion. Imaging and pathology slides are reviewed.

## Introduction

*Histoplasma capsulatum* is a dimorphic fungus that is endemic in many parts of the world including North, Central and South America. In the United States, it is most prevalent in the Ohio and Mississippi River Valleys. It grows well with humid conditions in acidic soil, with growth accelerated in sites contaminated with bird and bat excrement. Disturbance of the soil results in aerosolized spores that can travel over miles. Histoplasmosis can present with a wide range of symptoms from asymptomatic to life-threatening. Severity of illness after inhalation depends on the intensity of exposure and the immune status of the host. The majority of cases will resolve without therapy. Pulmonary manifestations are the most common symptomatic presentation [1].

## Case Report

A 19-year-old previously healthy Hispanic female presented with chief complaint of progressively worsening shortness of breath and heart palpitations for 1 week. She had previously recently been evaluated at an outside hospital without imaging and was diagnosed with gastritis, then discharged home. Prior medical history was positive for childhood asthma and removal of a lipoma from her right flank. There was no family history of heart disease, but she did note a paternal aunt with a history of unspecified mediastinal cancer. The patient emigrated to the United States from Mexico 2 years ago and had visited Mexico 2 months prior to presentation. She had never been pregnant and was not sexually active. She denied smoking, alcohol or drug use. On presentation, her vital signs were blood pressure 128/84, pulse 111 beats per minute, respiration rate 12 breaths per minute, and oxygen saturation 100% on room air. She appeared comfortable. Cardiovascular exam revealed normal rate and rhythm, grade 1 mid-frequency ejection murmur at left upper sternal border, normal upper extremity pulses and decreased lower extremity pulses. Blood pressure of right arm was 140/88, left arm was 136/90, right leg was 105/67 and left leg was 82/58. The remainder of the physical exam was normal.

**Figure 1:**
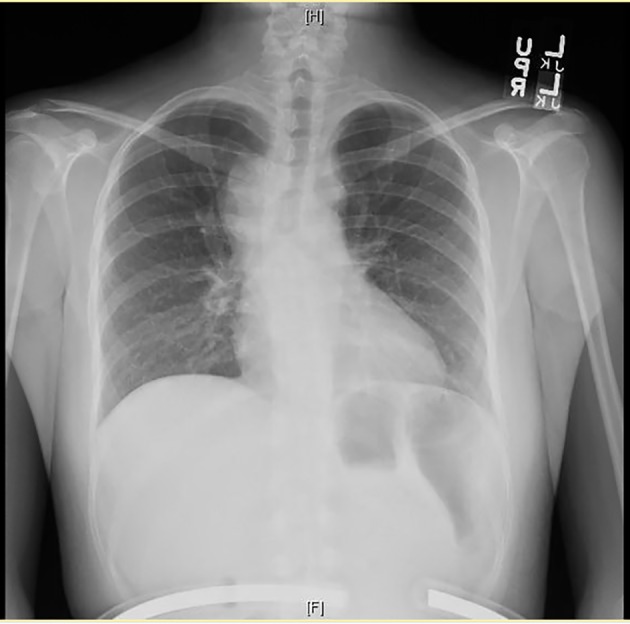
Chest X-ray showing right peri-hilar mass with tracheal compression.

**Figure 2:**
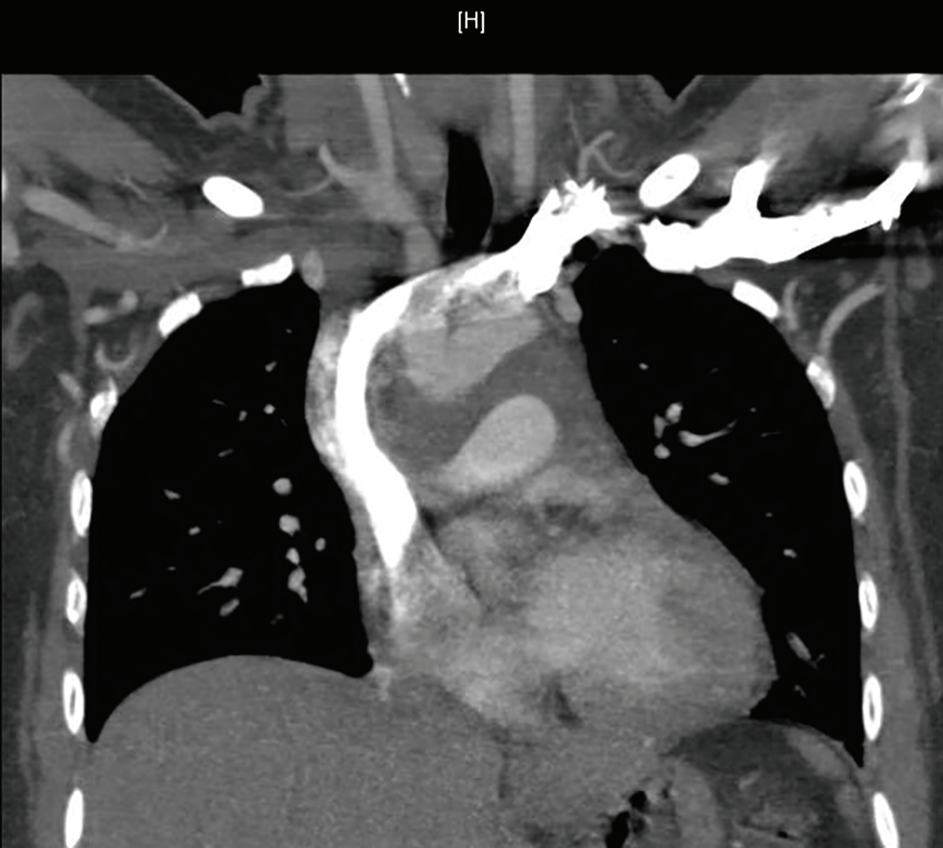
Computed tomography (CT) of chest showing mediastinal mass and mediastinal lymphadenopathy.

**Figure 3:**
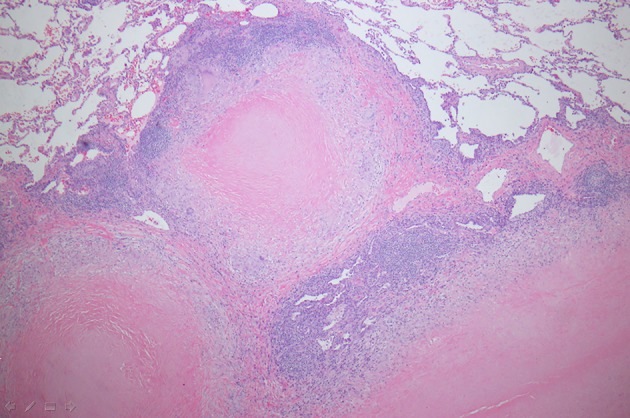
Wedge resection of right middle lobe of lung showing coalescing fibrocaseous granulomas (hematoxylin-eosin stain, magnification, × 100).

**Figure 4:**
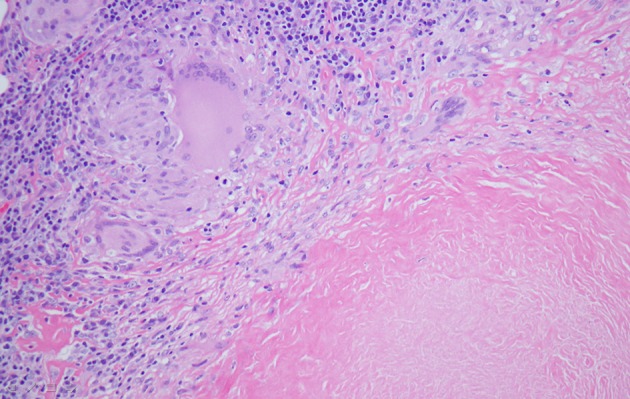
Wedge resection of right middle lobe of lung depicting giant cell histiocytes, epithelioid histiocytes, lymphocytes and fibrocaseous necrosis (hematoxylin-eosin stain, magnification, × 100).

**Figure 5:**
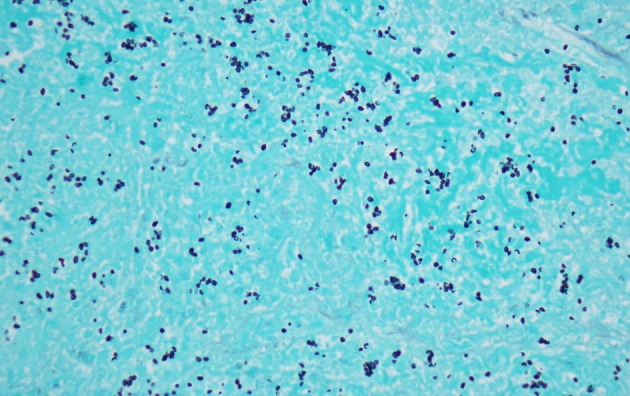
Wedge resection of right middle lobe of lung, magnification of fibrocaseous necrosis center of granuloma with budding yeast consistent with histoplasmosis species (Grocott methenamine silver stain, magnification, × 400).

Chest X-ray showed a right peri-hilar mass with some tracheal compression (Fig. 1). Computed tomography (CT) scan revealed a 1.2 × 1.1 cm mass in the right hilar region with associated diffuse mediastinal lymphadenopathy (Fig. 2). A previously undiagnosed severe isthmic coarctation of the aorta was noted immediately distal to the origin of the left subclavian artery with surrounding collateral arteries. Coarctation was confirmed with echocardiogram that showed peak gradient around 40 mm Hg. Systolic function was normal. Initial imaging was suspicious for malignancy or infectious process. There was concern that the coarctation may be related to the mediastinal process. Initial bronchoscopy with bronchoalveolar lavage (BAL) and fine-needle aspiration (FNA) were non-diagnostic. Subsequent endobronchial ultrasound (EBUS) with trans-bronchial needle biopsy of the mediastinal lymph nodes showed coalescing fibrocaseous granulomas with necrosis (Fig. 3, 4). Gram stain, culture and acid-fast bacilli (AFB) stain were negative. QuantiFERON gold test was also negative. Serology was positive for Histoplasma (titer 1:256). Pathologic analysis showed necrotizing granulomas with small fungal yeast that was morphologically most consistent with Histoplasma (Fig. 5).

**Figure 6:**
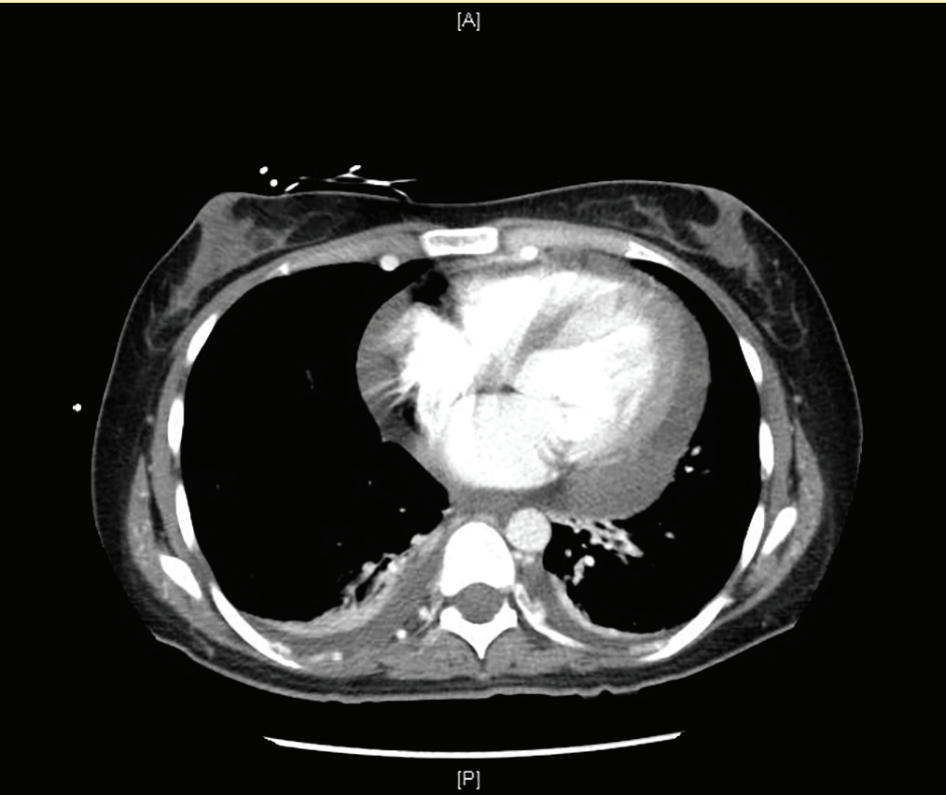
Computed tomography (CT) of chest showing pericardial effusion with pericardial thickening.

Repeat CT chest revealed pericardial thickening with massive pericardial effusion (Fig. 6). There was 2 cm pericardial thickening that was confirmed with echocardiogram. Emergent pericardiocentesis was required and 260 cc of yellow blood-tinged fluid was removed, then a pericardial drain was placed. The patient was treated initially with amphotericin B. Due to allergic reaction, she was transitioned to voriconazole. Surgical repair of the coarctation of the aorta was delayed until resolution of the Histoplasma infection. She eventually underwent surgical coarctectomy with end-to-end anastomosis with good results.

## Discussion

Pulmonary manifestations are the most common symptomatic presentation of histoplasmosis [1]. Several other authors have presented cases of histoplasmosis pericarditis [2-9]. Pericarditis is a known complication of pulmonary histoplasmosis, and has been reported in up to 5-10% of symptomatic patients [2-4]. Patients may present with a range of symptoms including fever, headache, myalgia, cough, chest pain, respiratory failure or death. The vast majority of symptomatic cases show pulmonary infiltrates with mediastinal lymphadenopathy. Pericarditis from histoplasmosis is generally attributed to an inflammatory response rather than an infectious process within the pericardium [4]. Treatment is indicated for moderate to severe acute or chronic pulmonary, disseminated and central nervous system histoplasmosis. Treatment is generally initiated with amphotericin B intravenously, followed by oral itraconazole. In patients with mild manifestations, treatment is usually unnecessary. Histoplasma pericarditis is recommended to be treated with non-steroidal anti-inflammatory (NSAID) therapy in mild cases [1]. It is unclear if antifungal therapy alters the course of histoplasmosis pericarditis due to lack of clinical trials [3].

Review of literature regarding histoplasmosis pericarditis did not reveal any previously published reports in conjunction with aortic coarctation. The aortic coarctation in this case is believed to be congenital rather than related to the histoplasma infection. However, the coarctation likely contributed to the severity of presentation. Here we provide a review of imaging showing an active pulmonary Histoplasma infection complicated by pericardial effusion in conjunction with aortic coarctation for the first time.
